# Relative Entropy Application to Study the Elastoplastic Behavior of S235JR Structural Steel

**DOI:** 10.3390/ma17030727

**Published:** 2024-02-03

**Authors:** Marcin Kamiński, Michał Strąkowski

**Affiliations:** Department of Structural Mechanics, Faculty of Civil Engineering, Architecture and Environmental Engineering, Łódź University of Technology, 90-924 Łódź, Poland; michal.strakowski@p.lodz.pl

**Keywords:** stochastic finite element method, Ramberg–Osgood material model, stochastic perturbation technique, Monte Carlo simulation, semi-analytical method, least squares method

## Abstract

The main issue in this work is to study the limit functions necessary for the reliability assessment of structural steel with the use of the relative entropy apparatus. This will be done using a few different mathematical theories relevant to this relative entropy, namely those proposed by Bhattacharyya, Kullback–Leibler, Jeffreys, and Hellinger. Probabilistic analysis in the presence of uncertainty in material characteristics will be delivered using three different numerical strategies—Monte Carlo simulation, the stochastic perturbation method, as well as the semi-analytical approach. All of these methods are based on the weighted least squares method approximations of the structural response functions versus the given uncertainty source, and they allow efficient determination of the first two probabilistic moments of the structural responses including stresses, displacements, and strains. The entire computational implementation will be delivered using the finite element method system ABAQUS and computer algebra program MAPLE, where relative entropies, as well as polynomial response functions, will be determined. This study demonstrates that the relative entropies may be efficiently used in reliability assessment close to the widely engaged first-order reliability method (FORM). The relative entropy concept enables us to study the probabilistic distance of any two distributions, so that structural resistance and extreme effort in elastoplastic behavior need not be restricted to Gaussian distributions.

## 1. Introduction

Structural materials and engineering structures exhibit various uncertainty sources ranging from environmental actions (as mechanical and/or thermal loadings) to material statistical imperfections, as well as some initial or manufacturing inaccuracies resulting in the necessity of reliability assessment [1]. This can be achieved on either a structural level or on the given material level, which is especially remarkable in the composite materials area but occurs also in classical civil engineering materials like structural steels. The situation is complex in the case of metallic materials due to their apparent nonlinearity under mechanical loads and the exploitation temperature variations on the one hand, and the need for the cross-section optimization on the other. This leads to the frequent application of nonlinear constitutive laws like Ramberg–Osgood having numerous applications [2,3,4,5,6,7,8,9,10,11,12], which can also be applicable in soil engineering [13]. Alternatively, one may employ Johnson–Cook, especially convenient for higher temperatures (traditional and modified formulations) [14], or Gurson–Tvergaard–Needleman [15] (including porosity in metal microstructures) in the modern finite element method (FEM) [16] simulations calibrated to the experimental data. Some more advanced modern theories include fractional plasticity models [17]. 

The major benefit of the Ramberg–Osgood model is the fact that it has a single parameter needing experimental verification, so its usage seems to be very efficient, not only in the field of steel structures but also in geotechnical applications. Such a nonlinear analysis becomes very complex where any uncertainty sources appear in the model—they can follow material micro, nano, or molecular scales defects, but also more classical statistical discrepancies in structural elements lengths, the location of the openings, holes, welds, or some inclusions [18,19]. Mechanical properties’ randomness in structural steel elements experimentation is most frequently noticed in the Young modulus and yield strength, which directly follow the unidirectional extension of the series of specimens. The standard deviations obtained for the brand-new elements are remarkably smaller than for the specimens cut from the extensively exploited structural elements under supervision, where additional corrosion processes may destroy both the geometry and material structure. 

It is well known that several mathematical and numerical methods have been established in engineering mechanics to model the aforementioned phenomena and to predict their structural impacts. Close to the analytical calculus of the basic probabilistic parameters [20], Bayesian approach [21], and Monte Carlo simulations family [22], one may find Karhunen–Loeve or polynomial chaos expansions [23], some semi-analytical techniques [20], as well as the group of stochastic perturbation methods [20,24]. The latter is formulated using various orders’ approaches as the first, the second, the third, or general order Taylor expansions leading to the determination of the first two, three, or four basic statistics of structural behavior. A general limitation of all of these techniques mentioned above resulting in the response statistics is a necessity of discussion of many parameters at the same time, which can be difficult and misleading. The alternative way is to directly investigate a failure danger by its probability, which remains less useful in practical engineering reliability assessment; one solution to this problem may be the determination of probabilistic entropy, whose fluctuations in mechanical problems enable uncertainty propagation discussion. More popular models include the Shannon [25], Renyi [26], and Tsallis [27] models together with their further modifications and improvements. 

Quite a similar situation takes place in reliability and durability studies, where the moment methods prevail [28]. The first-order reliability method (FORM) [29] convenient mostly for linear limit functions has been replaced over time by the second-order methodology (SORM) [30], and even by the first-order [31] or the second-order third moment (SOTM) technique [32]. The main limitation of both FORM and SORM is that they may be efficient in all of these situations, where the basic probabilistic moments are representative of the entire probability distribution of structural resistance or its extreme effort. Otherwise, Monte Carlo simulation or some alternative approach based on the probability density functions needs to be applied. The very interesting opportunity in this context is the so-called probabilistic divergence (relative entropy) [33], which enables quantifying with some single real value a distance in-between two random distributions while having their probability distributions or the basic probabilistic characteristics [34]. Various entropy measures for the uncertainty quantification of stochastic processes have been discussed in [35]. 

Unfortunately, a comparison between FORM and SORM and relative entropies apparatus is rather scarce in the literature, which mainly follows the remarkable number of totally different mathematical concepts in that area. Nevertheless, relative entropy application seems to be more adequate because the final formula is sensitive to the probability distribution type of both structural resistance and effort inherent in the limit function unlike in the FORM or SORM.

This study aims to achieve reliability assessment in the well-known tension test of the structural steel specimen, which is numerically simulated using the Ramberg–Osgood stress–strain relationship implemented in the FEM system ABAQUS. Probabilistic analysis has been carried out here using polynomial functions relating extreme deformations and von Mises reduced stresses with the Young modulus of the given steel type recovered numerically from several FEM tests with varying values of this parameter. Then, three different probabilistic methods, namely the stochastic perturbation technique, semi-analytical method, as well as the Monte Carlo simulation scheme, have been engaged to determine the first four probabilistic coefficients of the structural response. Finally, the first two of them have been used to calculate the reliability indices according to the FORM technique, and also alternatively—thanks to the application of the Bhattacharyya [36], Hellinger [37], Jeffreys [38,39], and Kullback–Leibler [40,41] relative entropies. As has been documented, classical FORM analysis may find its efficient alternatives in the modern computer-aided engineering of steel details and structures. The results obtained here for Gaussian input uncertainty may also be extended towards non-Gaussian distributions [42] with minor modifications of the numerical apparatus. 

## 2. Theoretical Background

### Governing Equations

The following incremental boundary value problem is considered in a certain solid body domain Ω having a continuous and sufficiently smooth boundary ∂Ω [43]:

(1) $$\Delta \sigma_{kl,l}+\rho \Delta f_{k}=0;\;x\in \Omega$$

(2) $$\Delta {\tilde{\sigma }}_{kl}=C_{klmn}\Delta \varepsilon_{mn};\;x\in \Omega$$

(3) $$\Delta \varepsilon_{kl}=\frac{1}{2}\left\{\Delta u_{k,l}+\Delta u_{l,k}+u_{i,k}\Delta u_{i,l}+\Delta u_{i,k}u_{i,l}+\Delta u_{i,k}\Delta u_{i,l}\right\};\;x\in \Omega$$

for *i, j*, *k*, *l* = 1, 2, 3 with the following boundary conditions:

(4) $$\Delta \sigma_{\bar{k}l}n_{l}=\Delta t_{\bar{k}};\;x\in \partial \;\Omega_{\sigma },\;\bar{k}=1,2,3$$

(5) $$\Delta u_{\hat{k}}=\Delta {\hat{u}}_{\hat{k}};\;x\in \partial \;\Omega_{u},\;\hat{k}=1,2,3$$

This problem is solved for the displacement vector $u_{k}\left(x\right)$, the strain tensor $\varepsilon_{kl}\left(x\right)$, and the stress tensor $\sigma_{kl}\left(x\right)$, the symbol Δ denotes their increments, while the fourth-rank tensor *C_klmn_* denotes here a constitutive tensor. This solution is achieved using the appropriate incremental version of the potential energy functional, and their minimization with respect to the displacement vector. Classical FEM discretization for nonlinear problems has been proposed with many numerical illustrations [44]. The entire probabilistic approach is based upon deterministic series of solutions of the iterative deterministic FEM equation as follows [45]

(6) $$K^{\left(m\right)}\Delta q^{\left(m\right)}=\Delta Q^{\left(m\right)}$$

where K denotes the stiffness matrix and ΔQ is the nodal loads’ increments vector, whereas Δq stands for the displacements vector increments; *m* indices here represent a current FEM test number necessary for the response function method recovery of polynomial bases. These bases are approximated via the least squares method (LSM) [46] from the series of the FEM experiments with some input parameters varying throughout their fluctuations ranges, which have been assumed a priori. 

A phenomenological constitutive model is analyzed here and it is known in the literature as the Ramberg–Osgood equation. It connects the unidirectional strain *ε* with the tensile stress σ using the following well-known formula:

(7) $$\varepsilon =\frac{\sigma }{E}+\alpha {\left(\frac{\sigma }{f_{y}}\right)}^{1/n}$$

where *f_y_* denotes the yield strength of the given material, *E* is its Young modulus, *α* corresponds to the yield effect, and *n* is the non-dimensional strain hardening coefficient. Quite naturally, *E* and/or *f_y_* may be perceived as some uncertainty sources because their mean values and other statistics result from strength experiments with well-documented statistics. However, derivation of any of the probabilistic analytical constitutive formulas would be rather difficult in this context as the uncertainty sources appear independently (or commonly) in the denominator of the aforementioned equation. 

**Figure 1:**
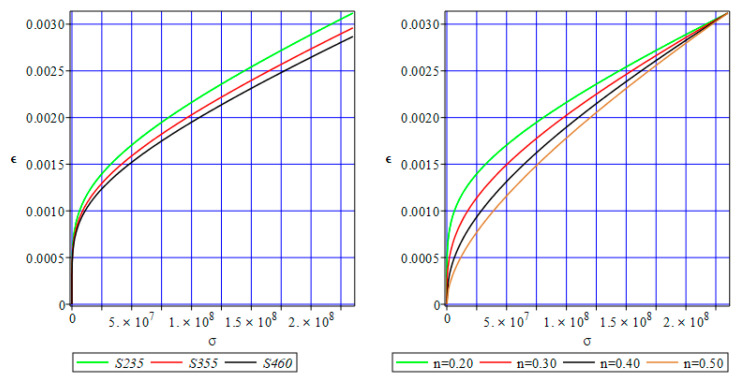
Stress–strain curves according to the Ramberg–Osgood law for different structural steels (**left**) and sensitivity of S235 to the strain hardening coefficient (**right**).

Visualization of the Ramberg–Osgood equation in its deterministic version is proposed below in Figure 1. This figure includes two graphs—the left one contains the stress–strain curves for three different structural sheets of steel, namely S235, S355, and S460, plotted for the material coefficients α = 0.002, which is a normative plastic strain according to Eurocode 3-1-1 and also with *n* = 0.1090 (adopted experimentally). The right graph is adjacent to the elastoplastic behavior of the weakest steel, S235, while modifying the strain hardening coefficient only. It is seen that the importance of this second parameter is more remarkable, especially in the early stages of deformation. The influence of the yield strength is generally smaller but is kept at almost the same level until the specimen failure. 

Generally, the elastoplastic analysis may lead to structural failure; therefore, reliability assessment gains a paramount importance. It engages the limit function relating the structural resistance *R* and extreme structural response *E* in the range of given boundary conditions. Further numerical analysis takes into account the serviceability limit state (SLS) function based upon the extreme and admissible displacements in the given specimen, and also the ultimate limit state (ULS) reduced von Mises stress and the corresponding yield strength of the structural steel under consideration. One may recall a definition of the reliability index due to the Cornell first-order theory [31]

(8) $$\beta \left(R,E\right)=\frac{E\left[R\right]-E\left[E\right]}{\sqrt{Var\left(R\right)+Var\left(E\right)-2Cov\left(R,E)\right)}},$$

which uses the first two probabilistic moments of both *R* and *E*, namely their expectations *E*[.] and variances *Var*(.). The determination of the expectations and variances of extreme displacements and extreme reduced stresses is conducted through a series of FEM experiments enabling the polynomial representation of these state functions concerning the given uncertainty sources. Such a polynomial basis is sought using the least squares method and enables further triple probabilistic calculus, where (i) its analytical integration with a probability kernel is the basis for the semi-analytical method, (ii) its Taylor series expansion leads to the stochastic perturbation scheme, and also (iii) random number generation and sequential processing of this polynomial statistical estimators. 

It is well-known that the FORM methodology has its limitations, so an alternative is frequently sought. Probabilistic divergence (entropy) between two different probability distributions may apply for this purpose, and Bhattacharyya theory contains the following to quantify the distance from *E* to *R* [34]:

(9) $$H_{B}\left(R,E\right)=\frac{1}{4}\frac{{\left(E\left[R\right]-E\left[E\right]\right)}^{2}}{\sigma^{2}\left(R\right)+\sigma^{2}\left(E\right)}+\frac{1}{2}\ln\left(\frac{\sigma^{2}\left(R\right)+\sigma^{2}\left(E\right)}{2\sigma \left(R\right)\sigma \left(E\right)}\right)$$

A comparison of these last two formulas exemplifies a need and suggests a way of upscaling the entropy *H*(*R*,*E*) to the FORM reliability index fluctuations level. There holds

(10) $$\beta =2\sqrt{\frac{1}{4}\frac{{\left(E\left[R\right]-E\left[E\right]\right)}^{2}}{\sigma^{2}\left(R\right)+\sigma^{2}\left(E\right)}+\frac{1}{2}\ln\left(\frac{\sigma^{2}\left(R\right)+\sigma^{2}\left(E\right)}{2\sigma \left(R\right)\sigma \left(E\right)}\right)}$$

It is seen that the entropy-based approach reduces to the FORM one when both uncertainty levels in *R* and *E* are equal to each other. This particular entropic approach follows a general formula given for two probability distributions of the variables *R* and *E*:

(11) $$H_{B}\left(p(R),p(E)\right)=\int_{-\infty }^{+\infty }{\left\{p\left(R\right)\left(x\right)p\left(E\right)\left(x\right)\right\}}^{\frac{1}{2}}dx$$

This model has been found useful in some previous reliability assessments for structural elasto-static and elasto-dynamic designing problems, but some referential models proposed by Kullback and Leibler [40], Jeffreys [39], and Hellinger [37] have been contrasted here.

(12) $$H_{KL}\left(p(R),p(E)\right)=-\int_{-\infty }^{+\infty }p(R)\left(x\right)\log\left(p(E)\left(x\right)\right)dx+\int_{-\infty }^{+\infty }p\left(E\right)(x)\log\left(p(R)\left(x\right)\right)dx$$

(13) $$H_{J}\left(p(R),p(E)\right)=H_{KL}\left(p(R)(x),p(E)(x)\right)+H_{KL}\left(p(R)(x),p(E)(x)\right)$$

(14) $$H_{SH}\left(p(R),p(E)\right)=\frac{1}{2}\int_{-\infty }^{+\infty }{\left(\sqrt{p(R)\left(x\right)}-\sqrt{p(E)\left(x\right)}\right)}^{2}dx=1-\int_{-\infty }^{+\infty }\sqrt{p(R)\left(x\right)p(E)\left(x\right)}dx$$

Similarly to the Shannon entropy definitions, these entropies have been introduced for the non-truncated Gaussian distribution, which may result in some small modeling error in some engineering problems, where structural parameters exhibit truncated character. Moreover, it can be demonstrated that these entropies in the case of two given different Gaussian probability distributions $p(R)\equiv N\left(E[R],\sigma (R)\right)$ $p(E)\equiv N\left(E[E],\sigma (E)\right)$ can be expressed in the following way (using their first two probabilistic moments only):

(i) Kullback–Leibler and Jeffreys’ relative entropies

(15) $$H_{KL}\left(p\left(R\right),p\left(E\right)\right)=\log\left(\frac{\sigma \left(E\right)}{\sigma \left(R\right)}\right)+\frac{\sigma^{2}\left(R\right)+{\left(E[R]-E[E]\right)}^{2}}{2\sigma^{2}(E)}-\frac{1}{2},$$

(16) $$\begin{array}{ll} H_{J}\left(p(R),p(E)\right) & =\log\left(\frac{\sigma \left(E\right)}{\sigma \left(R\right)}\right)+\frac{\sigma^{2}\left(R\right)+{\left(E[R]-E[E]\right)}^{2}}{2\sigma^{2}(E)}-1+\\ & \log\left(\frac{\sigma \left(R\right)}{\sigma \left(E\right)}\right)+\frac{\sigma^{2}\left(E\right)+{\left(E[E]-E[R]\right)}^{2}}{2\sigma^{2}(R)}\equiv symm\left(H_{KL}\left(p(R),p(E)\right)\right) \end{array}$$

(ii) the squared Hellinger relative entropy

(17) $$H_{H}\left(p(R),p(E)\right)=1-\sqrt{\frac{2\sigma (R)\sigma (E)}{\sigma^{2}(R)+\sigma^{2}(E)}}\exp\left(-\frac{1}{4}\frac{{\left(E[R]-E[E]\right)}^{2}}{\sigma^{2}(R)+\sigma^{2}(E)}\right)$$

Bhattacharyya entropy reduces in this case to Equation (9) given above.

## 3. Numerical Simulation of Uniform Extension of the Steel Cylinder

**Figure 2:**
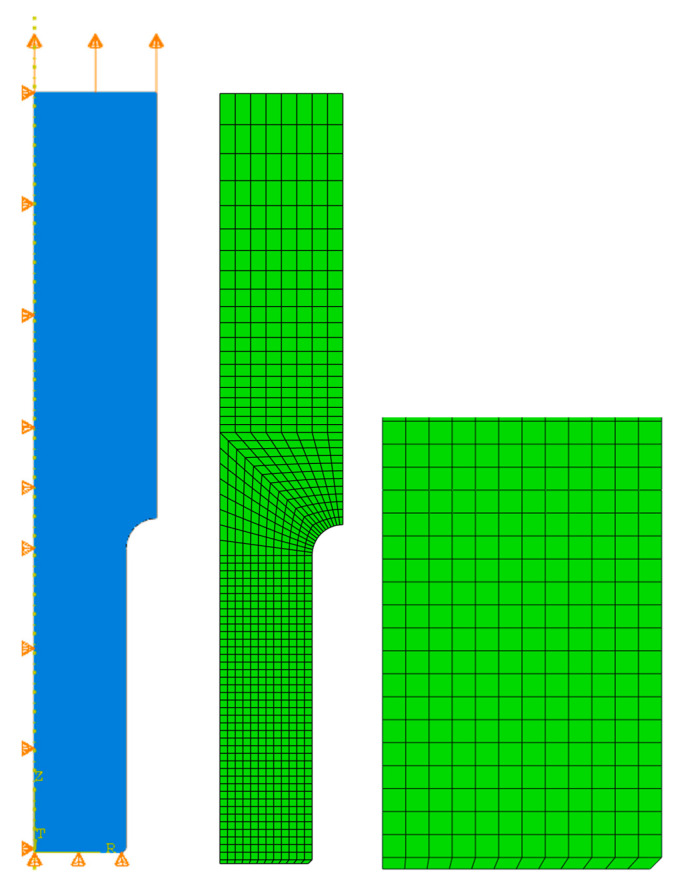
Geometry, boundary conditions, and meshing of the specimen.

A standard tensile test was carried out. Figure 2 shows the geometry of the specimen. Because of the axisymmetric cross-section of the round bar, only one plain section of it was conducted. To ensure necking appears, a small notch was made—this is a procedure well known from the literature [47]. In total, there are 844 quadrilateral FEM elements known in the ABAQUS system as CAX4R (4-node bilinear axisymmetric element). Reduced integration has been used in this case. As can be seen, the bottom part of the specimen was discretized with smaller FEM elements. The side of the brick element is 0.5 mm. The top part of the sample is divided using finite elements with different sizes. Close to the rounding in the middle of the height, the basic finite element size is 1.0 × 1.0 mm and is elongated to 1.0 × 2.0 mm on the top. It should be mentioned that some FEM studies are based on a combination of triangular (close to the necking) and quadrilateral elements (the remaining parts of the specimen) [15,16]. 

Figure 2 shows kinematic boundary conditions applied to the material specimen. The vertical displacements of the bottom edge are equal to zero (u_2_ = 0), whereas the horizontal displacements u_1_ = 0 for the left edge are the symmetry axes. The kinematic boundary conditions of the top edge have been provided as the extending load and are introduced here as u_2_ = 5.0 mm. The full Newton incremental method has been proposed to model incremental behavior in this case, and for this purpose, the initial increment size was assumed to be 0.001, the minimum equal to 0.0001, and the maximum allowed increment size was 1. Large displacements have been allowed in this case study. All numerical experiments with ABAQUS have been carried out in the Polish national network PL-Grid, and the approximate time consumption of the single incremental solution was about 5.0 min. 

**Figure 3:**
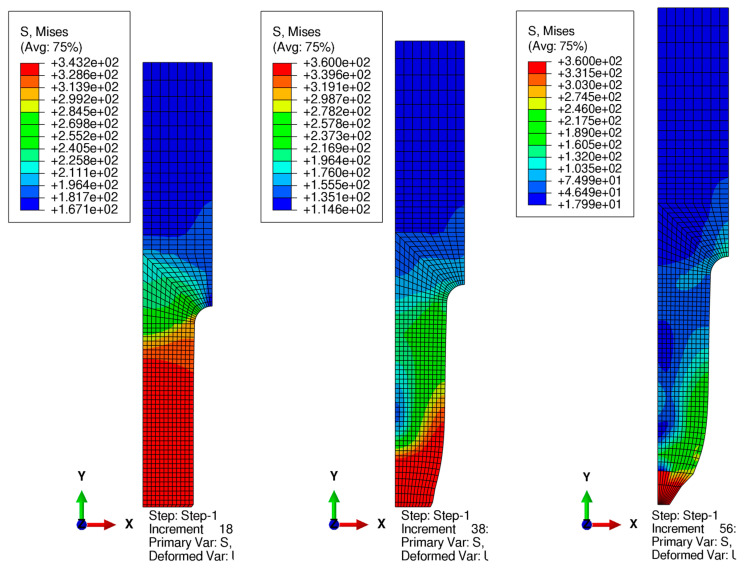
Resulting von Mises stress [MPa] distribution for 20, 60, and 100% of the analysis progress.

Figure 3 presents the resulting von Mises stress distribution in a few selected stages of the analysis (beginning, in the middle, and at its end). After 20% of the analysis (left drawing), the bottom part of the specimen has a stress close to the ultimate strength *f_u_* = 360 MPa here. After 50–60 percent of the analysis (middle drawing), necking occurs and the reduced stress reaches maximum values of 360 MPa. At the end of the analysis, necking is fully developed and the maximum stress is concentrated in the vicinity of this cross-section.

A further part of the numerical simulation has been delivered in the computer algebra system MAPLE, where the LSM approximation together with the three chosen probabilistic methods have been programmed. Additionally, the final part of the FORM and relative entropy computations have also been prepared in the same system by the script developed by the authors. Most of the results have been presented below as the functions of increasing uncertainty introduced in the model so that deterministic solutions are obtained as the lower bounds for all of these characteristics, and the largest scattering is the upper bound on the windows containing up to the fourth-order characteristics and entropies. 

**Figure 4:**
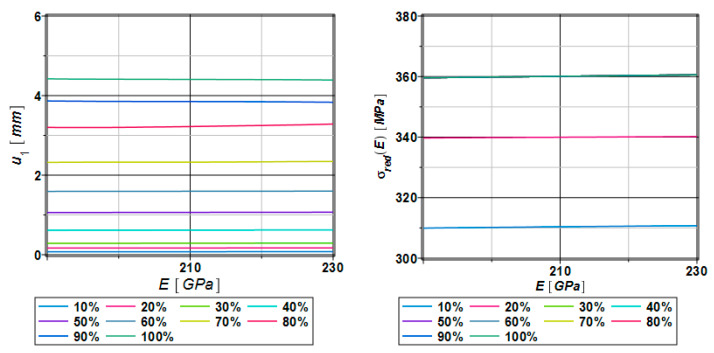
Response function polynomials of displacements and reduced stress as a function of the Young modulus E.

Figure 4 presents the response function polynomials of displacements (left one) dependent on Young modulus values in the range of 190, …, 210, …, 230 GPa. Such a relatively small dispersion causes displacements that are independent of the Young modulus. Moreover, the displacements are concentrated around its mean values. The right part of Figure 4 shows response function method polynomials through the entire Young modulus dispersion; whereas shortly after about 30% of the analysis progress, the resulting stress reaches maximum values of 360 MPa. All the resulting polynomials are independent of the Young modulus. Further, this parameter is adopted as the main uncertainty source in our specimen, which follows several experimental works in civil engineering. Its mean value of 210 GPa has been further considered as the expected value, while the standard deviation has been adopted as 10% of this value, which agrees with many laboratory verifications. The second motivation for this choice is that this parameter is inherent in most civil engineering reliability studies as the design parameter, so it can be decisive for designing the most advanced structures that need more attention. 

**Figure 5:**
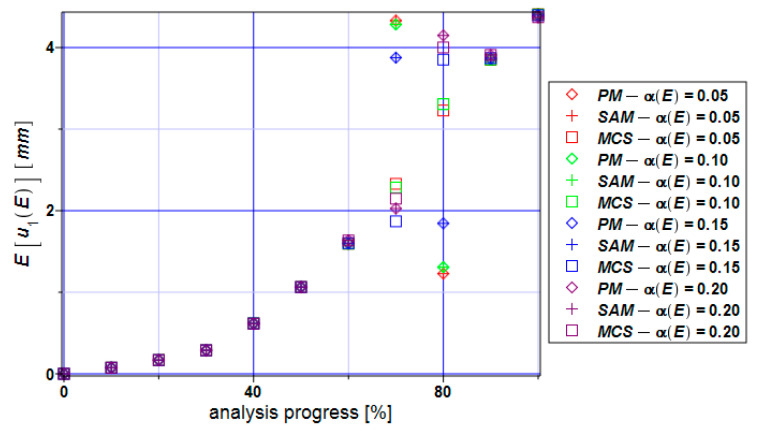
Expectations of the extreme displacements E[u_1_] for the specimen.

Figure 5 presents the distribution of the expectations of the extreme horizontal displacements as a function of the Young modulus E, and these displacements follow the range of the necking. The perturbation method (PM), semi-analytical method (SAM), and Monte Carlo simulation (MCS) have been compared. At the beginning of the analysis, each method provides the same values of the necking concentrated around the mean values. After about 70–80% of the process, some fluctuations can be observed. It can be identified with necking rapid expansion. 

**Figure 6:**
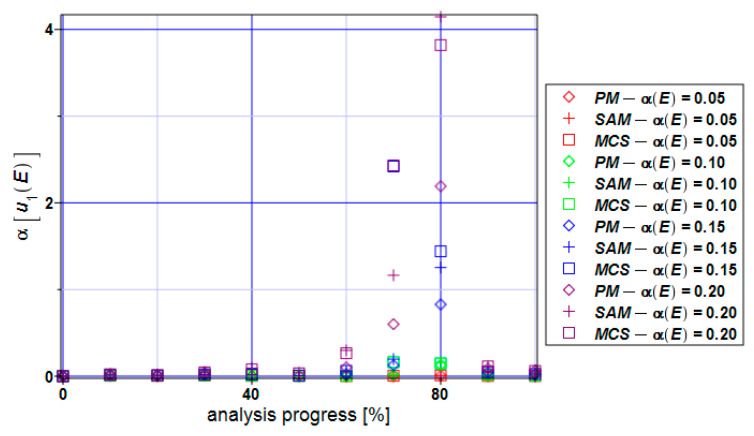
Coefficients of variation of the extreme displacements α[u_1_)] for the specimen.

Figure 6 shows the coefficients of the variation of the maximum horizontal displacements. Numerical values of this coefficient remain close to the input value throughout the entire analysis except for the extreme input uncertainty, which results in enormously large displacements of statistical scattering (even close to four, which never happens in elasticity). This extreme coefficient of variation is noticed for about 70–80%—the absolute extreme has been noticed while using the semi-analytical method, and a slightly smaller value has been detected with the use of the Monte Carlo simulation, whereas the perturbation method results in the minimum extreme value here.

**Figure 7:**
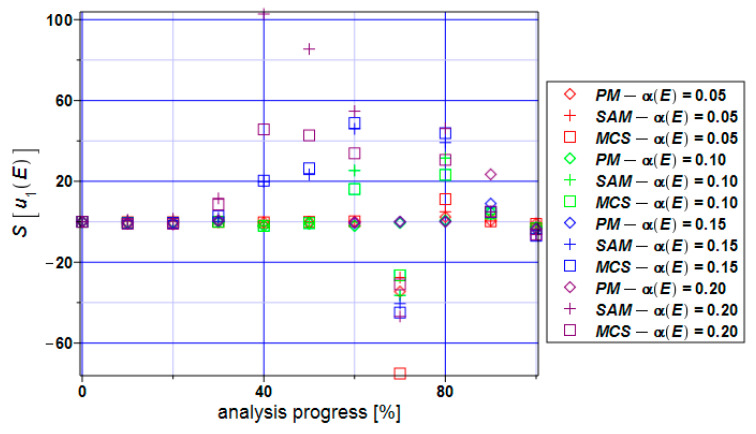
Skewness of the extreme displacements S[u_1_] for the specimen.

Figure 7 presents the distribution of the skewness values throughout the entire numerical experiment. At the beginning of this analysis, it takes values close to zero which tells us about the symmetry of the distribution of this state function. The skewness takes both positive and negative values for all three methods but most of them are positive. This tells us about the left-skewed distribution of the displacement state function. The skewness according to the perturbation method is close to zero if we narrow the input CoV α(E) < 0.15. 

**Figure 8:**
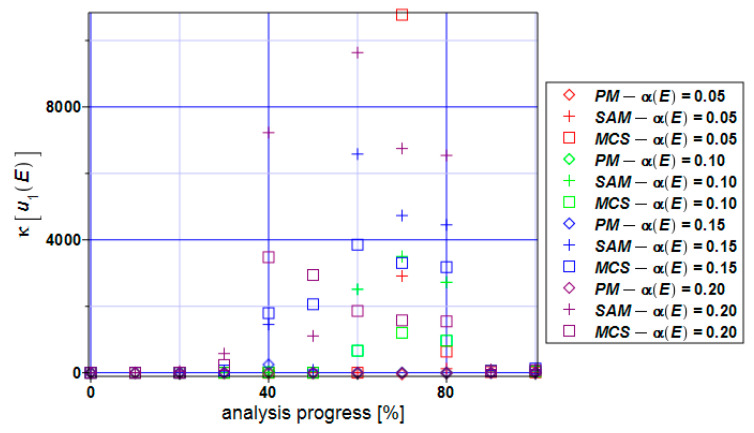
Kurtosis of the extreme displacements κ[u_1_] for the specimen.

The kurtosis of the horizontal displacements of the specimen (Figure 8) takes positive values according to all three methods. The perturbation method brings the values of the kurtosis closest to zero. The semi-analytical method and Monte Carlo simulation take positive values of more than 2000 in the middle part of the analysis if α(E) > 0.05. The biggest positive values that the kurtosis takes for the Monte Carlo simulation are for α(E) = 0.05. 

**Figure 9:**
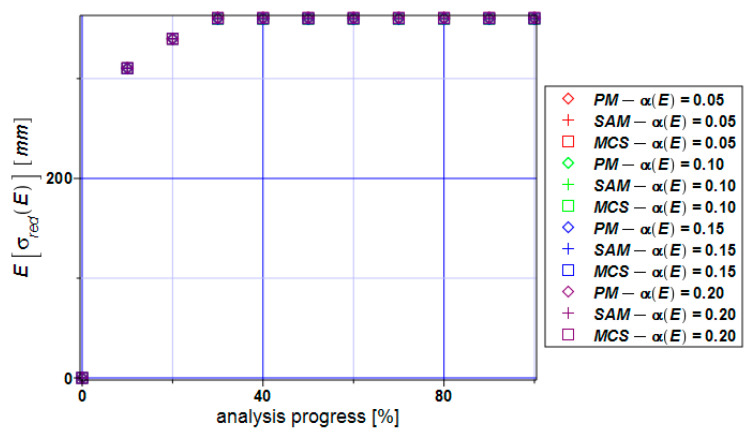
Expectations of the extreme reduced stress E[σ_red_] for the specimen.

Figure 9 shows expectations of the von Mises reduced stress for the specimen. It can be seen that all three methods bring the same values which are very close to their means in the whole range of the input CoV α(E) = [0.05,…,0.20]. 

**Figure 10:**
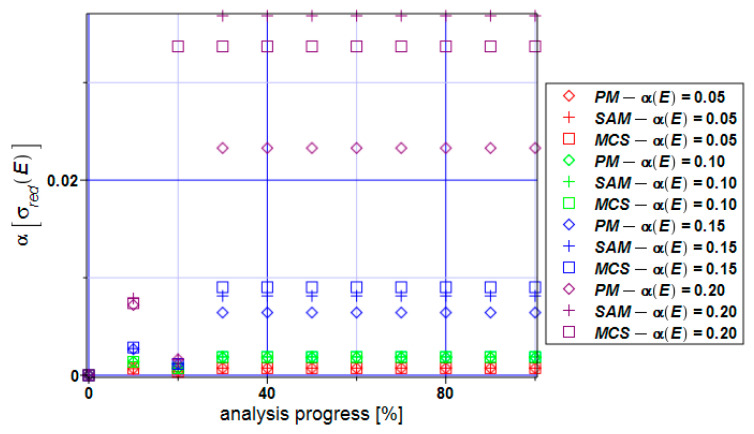
Coefficient of variation of the extreme reduced stress α[σ_red_] for the specimen.

The coefficient of variation of the reduced stress (Figure 10) is extremely close to zero based on all three methods. This means that the expectations reflect the mean values, and this fact can be seen in Figure 9. Practically no uncertainty is observed in the reduced stresses here while contrasting these results with the series from Figure 6. 

**Figure 11:**
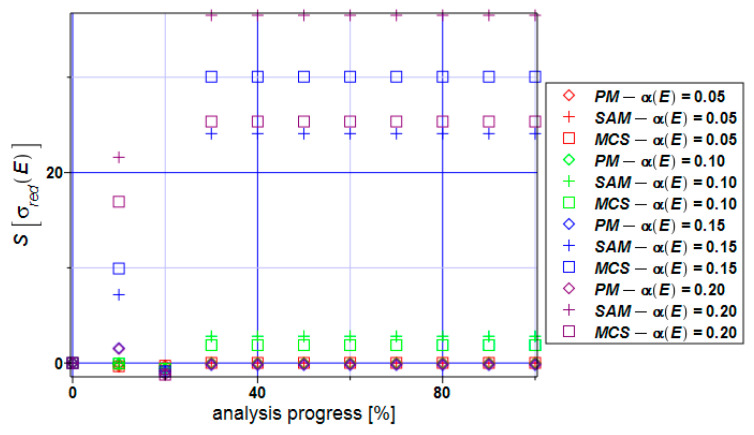
Skewness of the extreme reduced stress S[σ_red_] for the specimen.

The skewness contained in Figure 11 dominantly takes values close to zero if we limit the input coefficient of variation CoV to the values α(E) < 0.10. The semi-analytical method and Monte Carlo simulation bring values larger than 20 for the input CoV values 0.15 and 0.20. Positive values of the skewness tell us that the distribution of von Mises reduced stress is left-skewed and should not be modeled using Gaussian PDF.

**Figure 12:**
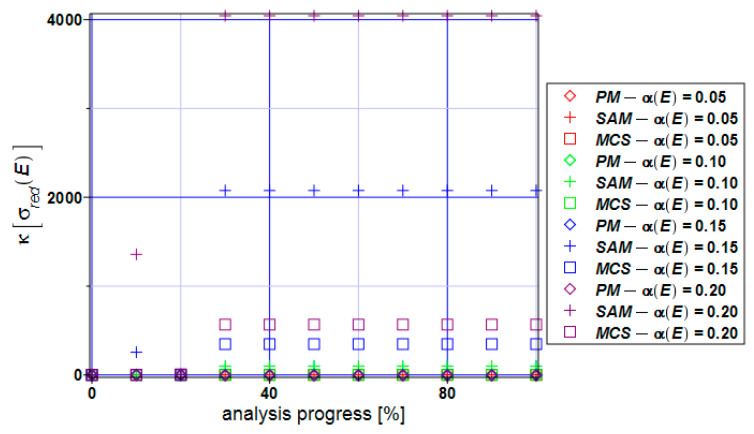
Kurtosis of the extreme reduced stress κ[σ_red_] for the specimen.

**Figure 13:**
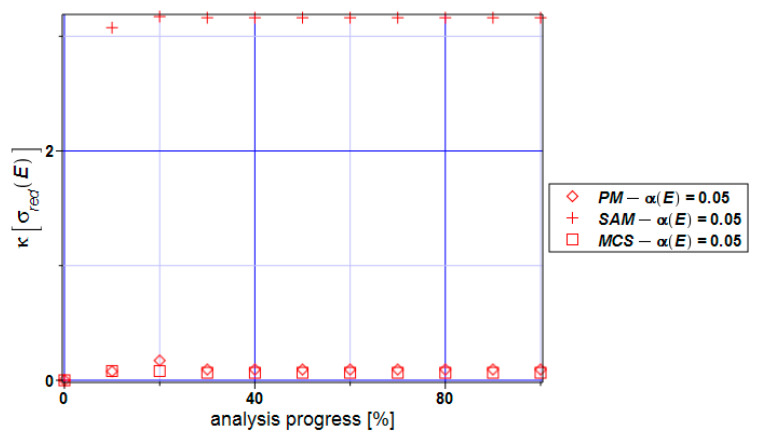
Kurtosis of the extreme reduced stress κ[σ_red_] for α(E) < 0.05.

The kurtosis of the extreme reduced stress (Figure 12) takes positive values throughout the entire extension process. The semi-analytical method and Monte Carlo simulation bring the values of the kurtosis into the range of 2000–4000 for α(E) > 0.10. This tells us that the von Mises reduced stress distribution has long tails and peaks. If one bounds the input CoV with α(E) ≤ 0.05 (Figure 13), then the distribution could be approximated by the Gaussian distribution with a relatively small modeling error. 

Figure 13 focuses on the kurtosis distribution if α(E) < 0.05. The perturbation method and semi-analytical technique bring values close to zero. Monte Carlo simulation provides a kurtosis close to three. 

**Figure 14:**
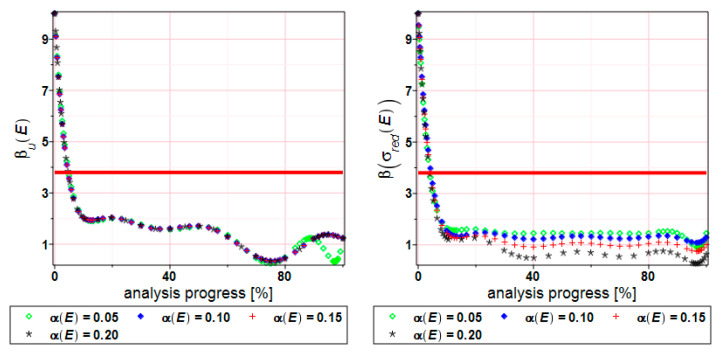
FORM reliability index of the displacements β_u_) (**left** graph) and extreme reduced stress β(σ_red_) (**right** graph) for the specimen.

Figure 14 shows the reliability index distribution regarding displacements (left) and stress state (right). The red line is a safety level for normal, typical constructions. The reliability index β_u_(E) takes the same values in a whole range of the input CoV. In total, 90% of the specimen width has been taken as a limit state for displacements. This is just an assumption because there is a complete lack of guidelines on how to calculate the limit value. It can be observed that the specimen reaches the failure region pretty fast (after 10% of the analysis progress). But it relates to the expected values of the reduced stress (Figure 9). Values equal to the ultimate strength 360 MPa can be noticed at the beginning of the process (after 30–40%). The stress of 360 MPa persists to the end of the analysis. The specimen is close to the breaking point, and that is the reason why the reliability index takes values below the red line. We can see that the reliability index depends on the input CoV α(E). The bigger dispersion of this value leads to smaller values of the index β(σ_red_(E)). Fluctuations in the reliability index of the reduced stress can be caused by RMS error according to the least squares method. Table 1 shows RMS error values, and one notices that the root mean square error dispersion is up to 100 times larger.

**Figure 15:**
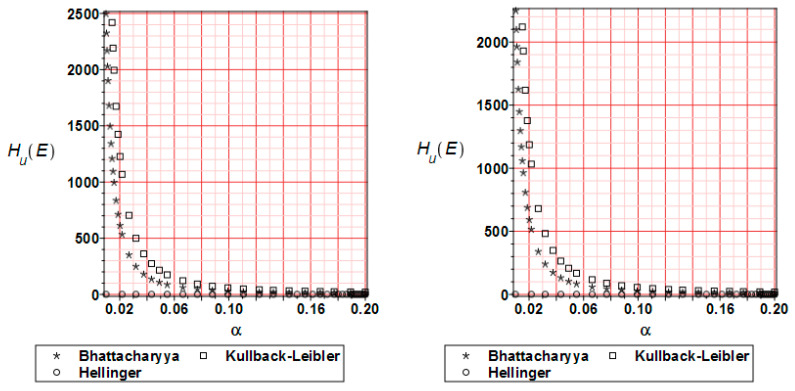
Relative entropy comparison for the displacements H_u_ at 20 (**left** graph) and 80% (**right** graph) of the analysis progress.

Figure 15 shows the relative entropy distribution for the horizontal displacements. The input CoV has been taken from the range α = [0,…,0.20]. Bhattacharyya, Kullback–Leibler, and Hellinger’s methods have been compared. During the whole analysis time, the first two methods bring similar results. In the beginning, the entropy reaches values over 2500 for α < 0.02. Then, it decreases to minimum values at the end of the analysis. It has to be underlined that Hellinger equations provide smaller values of the relative entropy. 

**Figure 16:**
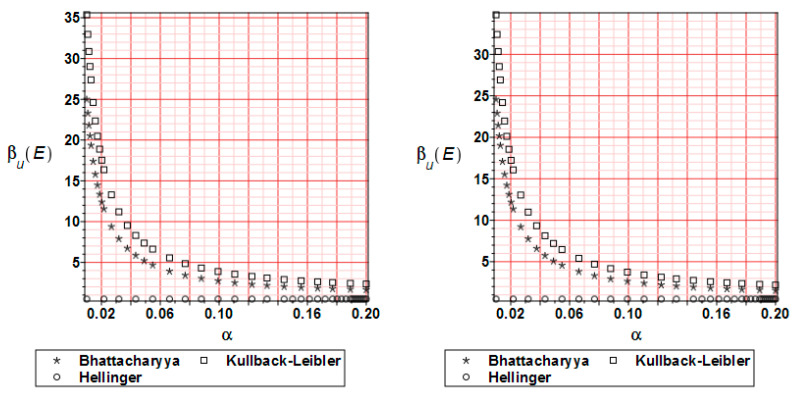
Reliability index β_u_ comparison for the displacements at 20 (**left** graph) and 80% (**right** graph) of the analysis progress.

Figure 16 presents the reliability index distribution for the displacements calculated based on relative entropy values. The nature of the curves is similar to Figure 15 but the values are proportionally smaller. Compared with Figure 14 (left), it can be observed that the reliability index takes values 4–5 times bigger.

**Figure 17:**
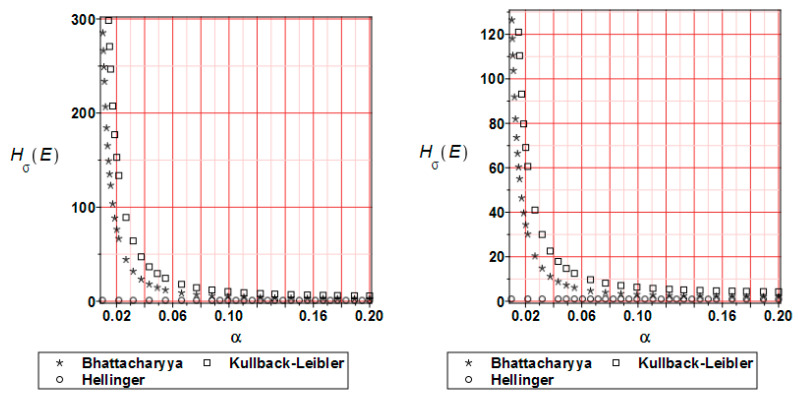
Relative entropy comparison for the reduced stress H_σ_ at 20 (**left** graph) and 80% (**right** graph) of the analysis progress.

The distribution of the relative entropy for the reduced stress H_σ_(E) after 20 and 80% of the analysis progress is presented in Figure 17. The most popular methods have been compared. As was observed for the displacements, Bhattacharyya and Kullback–Leibler methods bring similar character to the graphs; a larger input CoV causes a remarkable reduction of the entropy values. Hellinger’s method leads to values close to zero throughout the entire analysis. 

**Figure 18:**
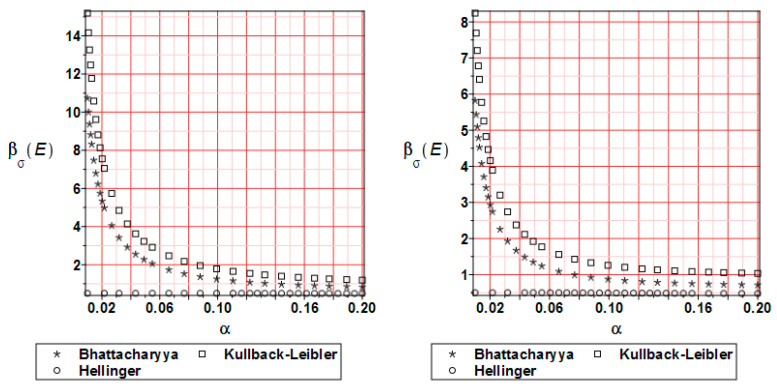
Reliability index β_σ_ comparison for the reduced stress at 20 (**left** graph) and 80% (**right** graph) of the analysis progress.

Figure 18 shows the reliability index distribution for the reduced stress. It is based on the relative entropy course. All curves are similar to the relative entropy distribution with the note that the reliability index takes smaller values by 10–15 times.

## 4. Concluding Remarks 

First, it has been demonstrated that the stochastic finite element method may be efficiently used in the determination of probabilistic characteristics up to the fourth-order of the structural response in computer simulation of nonlinear deformations governed by the Ramberg–Osgood material model. It can also be successively used in the reliability assessment when commonly applied with the first-order reliability method (FORM) or some alternative relative-entropy-based approach [48]. Secondly, the first four probabilistic moments of both extreme displacements and reduced stresses have been computed using three different methodologies, namely the generalized iterative stochastic perturbation technique, Monte Carlo simulation, and the semi-analytical method. The almost perfect agreement of all three methods for the first two probabilistic moments confirms the usefulness of this approach, guarantees the high quality of the results, and enables the alternative usage of all of these techniques when nonlinear problems with uncertainty are modeled. Common usage of the FEM system ABAQUS and computer algebra software MAPLE is recommended for such a hybrid computer analysis. The following conclusions can be formulated based on the presented example and its numerical results:

- Statistical dispersion of the input uncertainty on the level of 5% enables for treating structural responses inherent in the ULS and SLS as Gaussian, which may further simplify both the uncertainty analysis and the reliability assessment;
- The distribution of the reliability index β based on the relative entropy and relative entropy H have similar characters but their values differ by 10–20 times;
- The reliability index based on relative entropy according to the Bhattacharyya and Kullback–Leiber method for reduced stress brings numerical values quite close to the reliability index based on the FORM method;
- The Jeffreys and Hellinger’s approximation cannot be directly used for safety assessment.

Further work based on different steel structures is recommended to calibrate the reliability index while using the relative-entropy-based methodology to the limits proposed for the FORM index. It would also be interesting to apply the presented probabilistic apparatus in the numerical simulation of the reliability indices of the thermo-elasto-plasticity of structural steels, specifically in the context of dynamical loadings applied to the structure under consideration. 

## Figures and Tables

**Table 1:** RMS errors comparison.

Analysis Progress [%]	RMS Error
10	0.00129528
20	0.000244397
30	0.00147109
40	0.0000566029
50	0.0000711416
60	0.0000900214
70	0.00223297
80	0.00420256
90	0.00127888
100	0.000428241

## Data Availability

Data re contained within the article.

## References

[1] Melchers R.E. (2002). Structural Reliability Analysis and Prediction.

[2] Ramberg W., Osgood W.R. (1943). Description of Stress–Strain Curves by Three Parameters, Technical Note No. 902.

[3] Wei D., Elgindi M.B.M. (2013). Finite element analysis of the Ramberg-Osgood bar. Am. J. Comp. Math..

[4] Gadamchetty G., Pandey A., Gawture M. (2016). On Practical implementation of the Ramberg-Osgood Model for FE Simulation. SAE Int. J. Mat. Manuf..

[5] Anatolyevich B.P., Yakovlevna G.N. (2019). Generalization of the Ramberg–Osgood Model for Elastoplastic Materials. J. Mater. Eng. Perform..

[6] Elruby A.Y., Nakhla S. (2019). Extending the Ramberg–Osgood Relationship to Account for Metal Porosity. Metall. Mater. Trans. A.

[7] Skelton R.P., Maier H.J., Christ H.-J. (1997). The Bauschinger effect, Masing model and the Ramberg–Osgood relation for cyclic deformation in metals. Mater. Sci. Eng..

[8] Li J., Zhang Z., Li C. (2016). An improved method for estimation of Ramberg–Osgood curves of steels from monotonic tensile properties. Fatigue Fract. Eng. Mater. Struct..

[9] Niesłony A., el Dsoki C., Kaufmann H., Krug P. (2008). New method for evaluation of the Manson–Coffin–Basquin and Ramberg–Osgood equations with respect to compatibility. Int. J. Fatigue.

[10] Basan R., Fralunović M., Prebil I., Kunc R. (2017). Study on Ramberg-Osgood and Chaboche models for 42CrMo4 steel and some approximations. J. Constr. Steel Res..

[11] Kaldjiian M.J. (1967). Moment-Curvature of Beams as Ramberg-Osgood Functions. J. Struct. Div..

[12] Mostaghel N., Byrd R.A. (2002). Inversion of Ramberg–Osgood equation and description of hysteresis loops. Int. J. Non-Linear Mech..

[13] Papadimitriou A.G., Buckovalas G.D. (2002). Plasticity model for sand under small and large cyclic strains: A multiaxial formulation. Soil Dyn. Earthq. Eng..

[14] Wang X., Shi J. (2013). Validation of Johnson-Cook plasticity and damage model using impact experiment. Int. J. Impact Eng..

[15] Strąkowski M., Kamiński M. (2019). Stochastic Finite Element Method elasto-plastic analysis of the necking bar with material microdefects. ASCE-ASME J. Risk Uncertain. Eng. Syst. Part B Mech. Eng..

[16] Kossakowski P. (2012). The numerical modeling of failure of 235JR steel using Gurson-Tvergaard-Needleman material model. Roads Bridges.

[17] Qu P., Sun Y., Sumelka W. (2022). Review on Stress-Fractional Plasticity Models. Materials.

[18] Buryachenko V. (1999). Elastic-plastic behavior of elastically homogeneous materials with a random field of inclusions. Int. J. Plast..

[19] Kamiński M. (1999). Probabilistic characterization of porous plasticity in solids. Mech. Res. Commun..

[20] Kamiński M. (2013). The Stochastic Perturbation Method for Computational Mechanic.

[21] Hamada M.S., Wilson A.G., Reese C.S., Martz H.F. (2008). Bayesian Reliability, Springer Series in Statistics.

[22] Cardoso J.B., de Almeida J.R., Dias J.M., Coelho P.G. (2008). Structural reliability analysis using Monte Carlo simulation and neural networks. Adv. Eng. Softw..

[23] Ghanem R., Spanos P.D. (1991). Stochastic Finite Elements: A Spectral Approach.

[24] Kleiber M., Hien T.D. (1992). The Stochastic Finite Element Method.

[25] Shannon C.E. (1948). A mathematical theory of communication. Bell Syst. Tech. J..

[26] Renyi A. (1960). On measures of information and entropy. Proceedings of the Fourth Berkeley Symposium on Mathematical Statistics and Probability, Volume 1: Contributions to the Theory of Statistics.

[27] Tsallis C. (1988). Possible generalization of Boltzmann-Gibbs statistics. J. Stat. Phys..

[28] Zhao Y.-G., Ono T. (2001). Moment methods for structural reliability. Struct. Saf..

[29] Lopez R.H., Beck A.T. (2012). Reliability-Based Design Optimization Strategies Based on FORM: A Review. J. Braz. Soc. Mech. Sci. Eng..

[30] Cai G.Q., Elishakoff I. (1994). Refined second-order reliability analysis. Struct. Saf..

[31] Tichy M. (1994). First-order third-moment reliability method. Struct. Saf..

[32] Peng X., Geng L., Liyan W., Liu G.R., Lam K.Y. (1988). A stochastic finite element method for fatigue reliability analysis of gear teeth subjected to bending. Comput. Mech..

[33] Donald M.J. (1986). On the relative entropy. Commun. Math. Phys..

[34] Rauber T.W., Braun T., Berns K. (2008). Probabilistic distance measures of the Dirichlet and Beta distributions. Patt. Recognit..

[35] Namdari A., Li Z. (2019). A review of entropy measures for uncertainty quantification of stochastic processes. Adv. Mech. Eng..

[36] Bhattacharyya A. (1943). On a measure of divergence between two statistical populations defined by their probability distributions. Bull. Calcutta Math. Soc..

[37] Hellinger E. (1909). Neue Begründung der Theorie quadratischer Formen von unendlichvielen Veränderlichen. J. Reine Angew. Math. (Crelles J.).

[38] Jeffreys H. (1946). An invariant form for the prior probability in estimation problems. Proc. R. Soc. Lond. Ser. A Math. Phys. Sci..

[39] Nielsen F. (2021). Fast approximations of the Jeffreys divergence between univariate Gaussian mixtures via mixture conversions to exponential-polynomial distributions. Entropy.

[40] Kullback S., Leibler R.A. (1951). On information and sufficiency. Ann. Math. Stat..

[41] Teixeira R., O’Connor A., Nogal M. (2019). Probabilistic Sensitivity Analysis of OWT using a transformed Kullback-Leibler discrimination. Struct. Saf..

[42] Ghasemi S.H., Nowak A.S. (2017). Reliability index for non-normal distributions of limit state functions. Struct. Eng. Mech..

[43] Kleiber M., Woźniak C. (1991). Nonlinear Mechanics of Structures.

[44] Oden J.T. (1972). Finite Elements of Nonlinear Continua.

[45] Zienkiewicz O.C., Taylor R.C. (1989). The Finite Element Method.

[46] Bjorck A. (1996). Numerical Methods for Least Squares Problems.

[47] Aravas N. (1987). On the numerical integration of a class of pressure-dependent plasticity models. Int. J. Numer. Methods Eng..

[48] Zhang J., Pan Z., Zhang J., Bian J., Wang C. (2022). Rolling bearing state assessment based on the composite multiscale weight slope entropy and hierarchical prototype-based approach. Adv. Mech. Eng..

